# Process Mapping as an Innovative Method to Improve Uptake of Home Health Aide Services Among Veterans

**DOI:** 10.1093/geroni/igaf122.2758

**Published:** 2025-12-31

**Authors:** Lauren Hall, Margaret Ding, Bo Kim, Emily Franzosa

**Affiliations:** James J Peters VA Medical Center, New York, New York, United States; James J Peters VA Medical Center, New York, New York, United States; VA Boston Health Care System, Boston, Massachusetts, United States; Bronx VA, Bronx, New York, United States

## Abstract

The Veterans Health Administration (VA) offers a home health aide (HHA) benefit that supports older veterans’ desire to age in place. However, research from one VA medical center (VAMC) suggests only half of veterans referred to services take up the benefit. To understand reasons for this limited uptake, we conducted collaborative mapping of the process connecting veterans to HHA. Process mapping is widely used in healthcare settings to visualize steps in complex processes and identify areas for improvement. We conducted in-depth interviews with key informants of the HHA benefit at 5 VAMCs, including 15-20 primary care providers and HHA staff. We performed rapid analyses to identify process steps, barriers, and facilitators; mapped the elements using the online visual platform LucidChart; and standardized language across all VAMC-specific maps. We then held ‘report back’ sessions with key informants, to ensure the maps align with their firsthand understanding of the process. Salient barriers and facilitators confirmed by key informants included rurality’s impact on access and the VA teams’ commitment to connecting veterans to HHA services, respectively. Report back sessions also enabled expanded discussion and strategizing around identified best practices and implementation hurdles. New barriers identified in report-backs included capacity and resources of VA HHA program teams, and new facilitators included the benefit of adding a social worker perspective to the VA HHA team. Overall, this collaborative process enabled explicit delineation and shared understanding among key informants of their VAMC’s veteran-to-HHA connection.

